# Structural and functional insights into iron acquisition from lactoferrin and transferrin in Gram-negative bacterial pathogens

**DOI:** 10.1007/s10534-022-00466-6

**Published:** 2022-11-23

**Authors:** Clement Chan, Dixon Ng, Marie E. Fraser, Anthony B. Schryvers

**Affiliations:** 1 Department of Microbiology, Immunology and Infectious Diseases, Cumming School of Medicine, University of Calgary, Calgary, Canada; 2 Department of Biological Sciences, Faculty of Science, University of Calgary, Calgary, Canada; 3 Present Address: Department of Biochemistry, Faculty of Medicine, University of Toronto, Toronto, Canada

**Keywords:** Periplasmic binding protein, Bacterial iron transport, Transferrin, Lactoferrin

## Abstract

Iron is an essential element for various lifeforms but is largely insoluble due to the oxygenation of Earth’s atmosphere and oceans during the Proterozoic era. Metazoans evolved iron transport glycoproteins, like transferrin (Tf) and lactoferrin (Lf), to keep iron in a non-toxic, usable form, while maintaining a low free iron concentration in the body that is unable to sustain bacterial growth. To survive on the mucosal surfaces of the human respiratory tract where it exclusively resides, the Gram-negative bacterial pathogen *Moraxella catarrhalis* utilizes surface receptors for acquiring iron directly from human Tf and Lf. The receptors are comprised of a surface lipoprotein to capture iron-loaded Tf or Lf and deliver it to a TonB-dependent transporter (TBDT) for removal of iron and transport across the outer membrane. The subsequent transport of iron into the cell is normally mediated by a periplasmic iron-binding protein and inner membrane transport complex, which has yet to be determined for *Moraxella catarrhalis*. We identified two potential periplasm to cytoplasm transport systems and performed structural and functional studies with the periplasmic binding proteins (FbpA and AfeA) to evaluate their role. Growth studies with strains deleted in the *fbpA* or *afeA* gene demonstrated that FbpA, but not AfeA, was required for growth on human Tf or Lf. The crystal structure of FbpA with bound iron in the open conformation was obtained, identifying three tyrosine ligands that were required for growth on Tf or Lf. Computational modeling of the YfeA homologue, AfeA, revealed conserved residues involved in metal binding.

**Supplementary Information:**

The online version contains supplementary material available at 10.1007/s10534-022-00466-6.

## Introduction

Iron is an element that is essential for nearly all lifeforms to sustain crucial processes, such as energy production and oxygen transport. Despite its crucial roles, iron can be toxic to cells by catalyzing the formation of free radicals through the Haber–Weiss reaction (Kehrer 2000). It has been suggested that the abundance of ferrous iron in the primordial seas resulted in it being a preferred catalyst in biological redox reactions resulting in its essential role in various lifeforms today (Ostan et al. 2021). The gradual oxygenation of the Earth's atmosphere and oceans from 2.7 to 1.9 billion years ago led to the change of the atmosphere from a reducing to an oxidizing environment (Soo et al. 2017; Large et al. 2022; Sosa Torres et al. 2015). This caused soluble ferrous iron (Fe^2+^) to quickly oxidize into the insoluble ferric form (Fe^3+^) at a neutral pH, which rendered it inaccessible to many organisms. To access the insoluble iron, microbes produced and secreted iron binding molecules with increasing affinity and developed systems for the capture and uptake of the iron complexes. Over time this led to production of a diversity of different siderophores that are capable of ‘solubilizing’ iron so that it can be bound by its specific iron–siderophore receptor as the first step in the iron uptake pathway. In Gram-negative bacterial species this process is mediated by TonB-dependent transporters (TBDTs) that use energy derived from an inner membrane anchored complex (TonBExbBExbD) to transport iron across the outer membrane (Postle 1993).

Early metazoans initially developed single-lobe iron binding proteins to capture iron from sea water that are considered ancestral to the modern day bilobed iron-binding proteins in mammals, transferrin (Tf) and lactoferrin (Lf) (Williams 1982). Through gene duplication and the subsequent gene fusion that occurred between 850 and 670 million years ago, the primordial single lobe iron binding protein evolved into the bi-lobed Tf protein (Lambert et al. 2005; Park et al. 1985). Lf arose through a second gene duplication event in the mammalian lineage that occurred around 125 million years ago (Lambert et al. 2005; Lambert 2012). Using conventional terminology Tf and Lf would be considered fused dimers of two bi-lobed iron binding proteins but their recognition as a bi-lobed proteins is well entrenched in the literature.

The initial identification and isolation of host specific Tf and Lf receptors in the human pathogen *Neisseria meningitidis* (Schryvers and Morris 1988a, b) was followed by identification of host specific receptors in Gram-negative pathogens of humans and food production animals that reside exclusively in the upper respiratory or genitourinary tract of their mammalian host (Gray-Owen and Schryvers 1996; Morgenthau et al. 2013). Evidence for their importance for survival on the mucosal surface was provided by experiments with *Neisseria gonorrhoeae* in human male volunteers (Cornelissen et al. 1998; Anderson et al. 2003) and an aerosol infection model in pigs (Baltes et al. 2002). However, the specific demonstration that the Tf or Lf receptors are required for colonization of the upper respiratory tract by bacteria that reside exclusively in their mammalian host is currently lacking. The host specificity for human Tf by the bacterial receptor proteins has been shown to be due to selective pressures on Tf residues involved in binding to the bacterial receptor proteins that occurred during 40 million years of primate evolution (Barber and Elde 2014). By extension of this conclusion the presence of Tf receptors in birds (Ogunnariwo and Schryvers 1992) that have limited sequence identity to the mammalian Tf receptors, suggests that Tf has been available as a source of iron for growth on the mucosal surface for over 320 million years (Ostan et al. 2021).

**Fig. 1:**
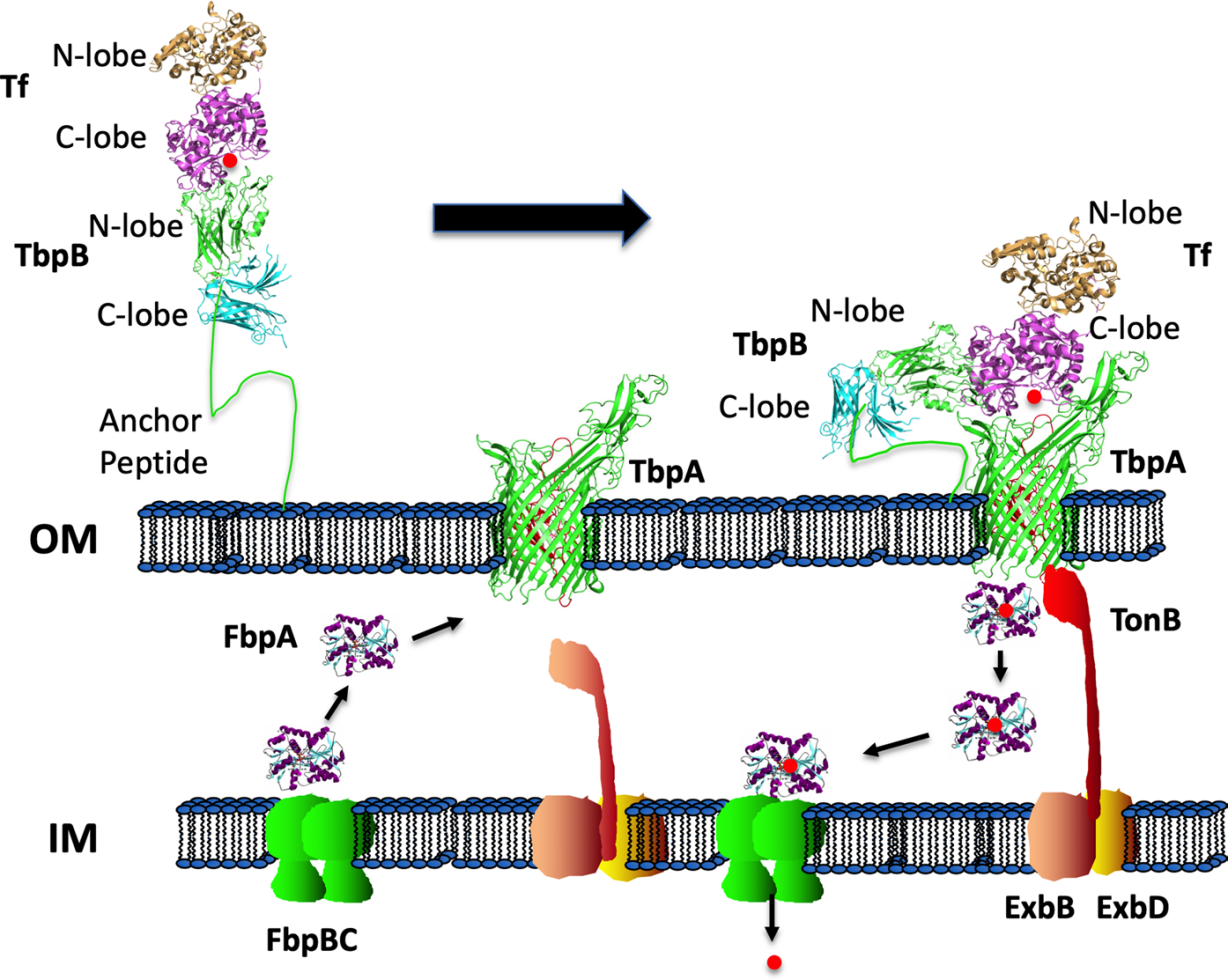
Iron acquisition from transferrin. Due to its long anchoring peptide TbpB extends from the surface of the outer membrane to capture iron loaded Tf. TbpB delivers the iron-loaded Tf to TbpA, triggering interaction with TonB that provides energy derived from the ExbB-ExbD-TonB complex for transporting the iron across the outer membrane. The iron released from Tf is then transferred to the periplasmic iron binding protein FbpA (ferric binding protein A) which shuttles across the periplasmic space and interacts with an inner membrane FbpB-FbpC transport complex that uses ATP hydrolysis to transport the ferric ion into the cytoplasm

The common bipartite Tf receptor consists of a TonB-dependent integral outer membrane protein, Tf binding protein A (TbpA), and a surface anchored lipoprotein, Tf binding protein B (TbpB) (Morgenthau et al. 2013) (Fig. 1). The TbpB surface lipoprotein (SLP) has an N-terminal anchor peptide region that allows extension away from the surface of the outer membrane to capture iron-loaded Tf (Moraes et al. 2009; Yang et al. 2011) and then promotes interactions with TbpA (Yang et al. 2011) to form a ternary TbpB:Tf:TbpA complex for iron removal. The domain separation in the C-lobe of Tf facilitates removal of iron that is then transported across the outer membrane (Noinaj et al. 2012a) in a process energized by interaction with TonB from the TonB-ExbB-ExbD complex (Fig. 1). The iron is transferred to the periplasmic ferric binding protein A (FbpA) that delivers it to an inner membrane transport complex (FbpBC) that transports the iron into the cytoplasm (Fig. 1).

The two component Lf receptor is similar to the Tf receptor (Fig. 1) and likely arose from a Tf receptor after Lf became available in mammals around 125 million years ago (Lambert 2012). Since Lf did not have to play an essential role in iron homeostasis, it acquired a host of additional specialized functions that are important for the control of microbial growth and disease prevention (Ostan et al. 2021). Its presence in mucosal secretions where it could effectively sequester available iron is likely responsible of the two component Lf receptor that clearly was derived from the Tf receptor in two bacterial lineages (*Neisseriaceae* and *Moraxellaceae* families). The development of a cationic N-terminal region capable of generating cationic antimicrobial peptides from Lf by proteolysis likely was the driving force for the development of the negatively charged loop regions present in the LbpBs that provide protection against these agents (Morgenthau et al. 2014).

As expected, the process of iron acquisition from Lf by the two component Lf receptor complex is similar to that of the Tf receptor although the presence of the negatively charged regions on LbpB have made the experimental studies more challenging (Morgenthau et al. 2013; Yadav et al. 2019). In *M. catarrhalis* and *Moraxella bovis*, previous experiments have shown that the deletion of an iron-regulated TBDT, CopB and IrpA respectively, interferes with the ability to grow on Tf and Lf as a sole source of iron (Chan et al. 2021; Kakuda et al. 2003; Aebi et al. 1996). The homologue in the pathogenic *Neisseria* species, FetA or FrpB, was originally identified as a siderophore receptor (Carson et al. 2000, 1999), but there have not been any reports on its impact on iron acquisition from Tf or Lf. However, insertional inactivation of the *fetA* gene and a downstream *fetB* gene, a presumed periplasmic siderophore binding protein (SBP), abolished utilization of an iron-enterobactin preparation as a source of iron for growth (Carson et al. 1999). FetA is one of eight TBDTs encoded in the gonococcal genome and is also found in the commensal *Neisseria* with the closest homologues being siderophore transporters (Cornelissen and Hollander 2011). Structural studies with FrpB (Saleem et al. 2013) clearly demonstrate that it directly binds to the iron atom which is not typical for many siderophore receptors and raises the question of whether it primarily transports iron. Similar to the pathogenic *Neisseria* species, the Tf and Lf receptors in *M. catarrhalis* have been identified and functionally characterized (Luke and Campagnari 1999; Bonnah et al. 1999) and the mechanism by which CopB affects iron acquisition from Tf and Lf has been shown to be indirect (Chan et al. 2021). To have a more complete understanding of this phenomenon and how it compares to the situation in *Neisseria* it would be important to compare the roles of the periplasmic binding proteins in these two species.

Ongoing structural and functional studies have identified a growing number of iron-binding periplasmic proteins such that genome annotation or BLAST searches can be used to identify putative iron binding periplasmic proteins (Murphy et al. 2016). However, this is only able to find putative homologues of identified iron-binding periplasmic binding proteins and if thorough structural and functional analysis is not performed it can lead to mistakes in annotation. For example, an early study identified a putative periplasmic iron binding protein in *Actinobacillus pleuropneumoniae* with limited experimental evidence (Chin et al. 1996) that was named AfuA. Subsequently, an AfuA protein was identified in *Aggregatibacter actinomycetencomitans* that had high similarity with FbpA (HitA) from *Haemophilus influenzae* and aligned with several other homologues including AfuA from *Actinobacillus pleuropneumoniae*. Attempts at crystallizing AfuA from *A. pleuropneumoniae* with bound iron were unsuccessful but a high-resolution structure of the protein revealed a bound sugar phosphate that ultimately led to studies demonstrating that this sugar phosphate binding protein contributes to the pathogenesis of *Citrobacter rodentium* (Sit et al. 2015). To date, the authentic ferric binding protein required for the uptake of iron from Tf in *A. pleuropneumoniae* has not been identified. Since the periplasm to cytoplasm iron acquisition pathways in *M. catarrhalis* had not been elucidated, the current study was initiated to identify and characterize these pathways and their impact on iron acquisition from Tf and Lf.

## Results

### Comparison of iron acquisition proteins in *Moraxella* and *Neisseria* species

Previous studies have described or compared the Tf and Lf receptors in *Neisseria* and *Moraxella* species (Ostan et al. 2021; Chan et al. 2018) but there is little information comparing the repertoire of other iron acquisition proteins in these genera. FetA in *Neisseria*, CopB in *M. catarrhalis* and IrpA in *M. bovis* are reasonable homologues of each other (44% identity, Table 1) and their structural models preserve the overall canonical features. The third extracellular loop contains a REEF domain at the beginning that has been shown to be important for iron acquisition from Tf and Lf in CopB (Chan et al. 2021). All three proteins have adjacent His and Tyr amino acid residues that are positioned for effective iron binding and were also shown to be important for iron acquisition from Tf and Lf by CopB (Chan et al. 2021). FetA and CopB differ in that it is loop 5 in FetA and loop 3 in CopB that vary substantially in size whereas structural models predict that it is loop 2 in IrpA that is particularly large (Fig. S1). Loop 5 in FetA has been proposed to shield critical immunogenic domains in FetA/FrpB (Saleem et al. 2013; Kortekaas et al. 2006).

**Table 1:** Presence of iron acquisition proteins in *Moraxella catarrhalis*, *M. bovis* and pathogenic *Neisseria*

Bacterial strain	CopB homologue	% Identity	AfeA homologue	% Identity	FbpA homologue	% Identity	FetB homologue	% identity
*Moraxella catarrhalis*	CopB	100	AfeA	100	FbpA	100	No	
*Moraxella bovis*	IrpA	44	Yes	79	no	–	Yes	46
*Neisseria gonorrhoeae*	FetA	44	MntC	29	FbpA	29	FetB	100
*Neisseria meningitidis*	FetA	44	No		FbpA	29	FetB	95

The periplasm to cytoplasm iron transport pathways in *M. catarrhalis* were initially identified by performing PSI-BLAST searches with known iron-binding periplasmic binding proteins as described in the methods section (Altschul et al. 1997). This approach only identified two iron-binding periplasmic proteins that were homologues to FbpA from the bovine pathogen *Mannheimia haemolytica* with 39% identity (Shouldice et al. 2003a, 2004) and to YfeA from *Yersinia pestis* with 70% identity (Bearden and Perry 1999). The YfeA homologue*,* AfeA, had previously been identified by an analysis of the genomic sequence of a single *M. catarrhalis* isolate (Murphy et al. 2016).

The *M. catarrhalis* FbpA was used in a PSI-BLAST search of the two pathogenic *Neisseria* species and was shown to only have 29% amino acid identity to well-characterized FbpA in those species (Table 1). The *Neisseria* FbpA is a close homologue of the protein from *H. influenzae* (Bruns et al. 1997) which was noted to have an iron co-ordination scheme nearly identical to Tf. Although *M. bovis* must have a periplasmic protein with a similar function, it cannot be identified by BLAST searches, highlighting the limitations of bioinformatics approaches for identifying this class of proteins. Since FetB was required for growth dependent on iron complexed with enterobactin (Carson et al. 1999) PSI-BLAST searches were performed with the FetB protein from *N. gonorrhoeae*/*N. meningitidis.* Although they did not identify any homologues in *M. catarrhalis*, a potential homologue with 46% identity was present in *M. bovis* (Table 1). PSI-BLAST searches with the *M. catarrhalis* AfeA protein identified a homologue in *M. bovis* with 79% identity, a potential homologue, MntC, in *N. gonorrhoeae* with only 29% identity and none in *N. meningitidis*. Thus, there does not appear to be a common pathway for transporting iron from FetA, CopB and IrpA to the cytoplasm.

### The function of *M. catarrhalis* FbpA and AfeA

**Fig. 2:**
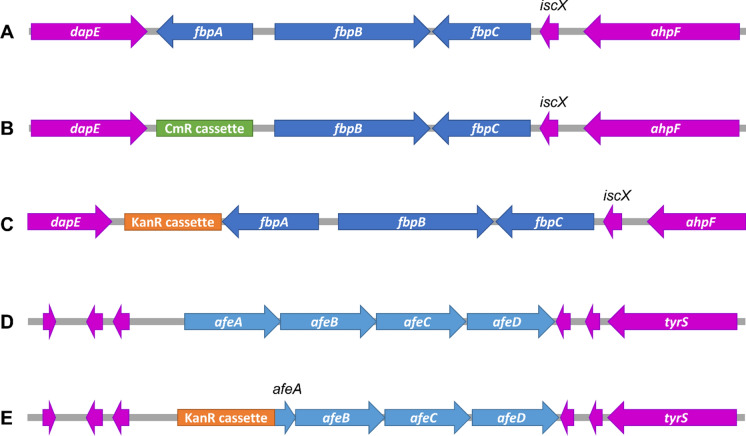
Schematic diagram of the *M. catarrhalis fbp* and *afe* loci. **a** *fbp* locus of the wildtype strain (N141). **b** *fbp* locus of the Δ*fbpA* mutant (N404). **c** Δ*fbpA* mutant complemented with wildtype or mutant *fbpA* in *cis* (N509, N515, N517, or N519). **d** *afe* locus of the wildtype (N141). **e** *afe* locus of the Δ*afeA* mutant (N473). The *M. catarrhalis fbp* operon is flanked by *dapE*, *ixcX*, and *ahpF*, whereas the *afe* operon is flanked by *tyrS*, and five unknown genes

To investigate the role of *M. catarrhalis* FbpA and AfeA in utilization of different iron sources, we created single gene knockout strains for both *fbpA* and *afeA* to be used for testing. A chloramphenicol acetyltransferase cassette or an aminoglycoside-3′-phosphotransferase gene was inserted between two ~ 600 bp regions that are upstream and downstream of the open reading frame via SOE-PCR, allowing homologous recombination with the bacterial genome (Fig. 2). This removed the entire gene of interest from the genome. We were able to obtain Δ*afeA* mutant colonies using BHI medium. In contrast, we were unable to obtain Δ*fbpA* mutant colonies with BHI but were only able to do so when using chocolate agar as the growth medium. A Δ*fbpA*Δ*afeA* mutant strain could not be produced using either growth medium.

After creating the knockout strains, we used a feeding assay where the iron chelator, Desferal, is added into the BHI agar media. Different strains were inoculated onto the agar plates and various iron sources (human Tf, human Lf, haemin, ferric citrate) were then spotted on to the plates. Porcine Tf, an iron source that *M. catarrhalis* is unable to utilize, was spotted as a control to test for the potential that the bacteria can survive on the iron released by a degraded iron carrier proteins.

**Fig. 3:**
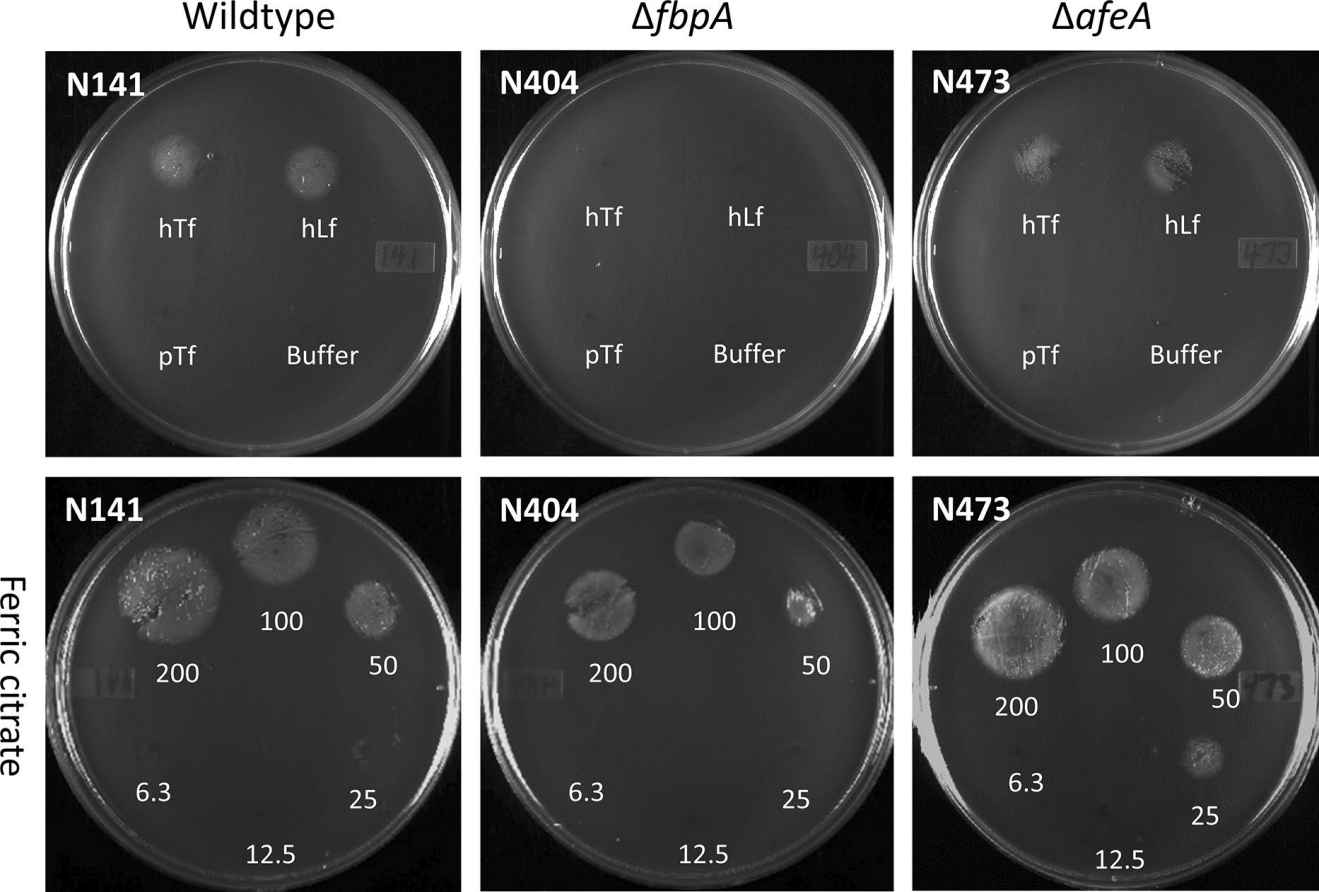
Feeding assay on BHI plates containing 50 μg/mL of Desferal. The plates were either inoculated with the wildtype strain (N141), the Δ*fbpA* mutant (N404), or the Δ*afeA* mutant (N473). For the top three panels, 3.1 nmol of hTf, hLf, pTf, and buffer was spotted onto the plate. For the bottom three panels, different dilutions of ferric citrate (200, 100, 50, 25, 12.5, and 6.3 nmol) were spotted onto the plate

The wildtype *M. catarrhalis* strain and the Δ*afeA* mutant strain were able to utilize human Tf and human Lf as iron sources (Fig. 3). In contrast, the Δ*fbpA* mutant was unable to grow on either human Tf or Lf. No growth was observed on porcine Tf for any of the strains. Although the wild-type strain and Δ*fbpA* mutant grew on ferric citrate, the knockout required a twofold higher concentration than the wild-type strain to result in visible growth (Fig. 3). All three strains grew on haemin (data not shown).

### Structural determination of *M. catarrhalis* FbpA

The *fbpA* gene from *M. catarrhalis* was cloned into our custom periplasmic expression vector resulting in the production of high levels of the *M. catarrhalis* FbpA (McFbpA) in the *E. coli* periplasm that was released by osmotic shock. The protein was purified by anion-exchange chromatography and then iron-loaded with a ferric citrate/bicarbonate buffer. The iron-loaded protein was concentrated to 50 mg/mL and was screened for crystallization conditions using the Hampton Index HT crystallization screen. Two crystallization conditions were identified within 1 week after setup, producing the apo (colourless) and the holo (red–orange) crystal forms. The apo McFbpA structure was solved via molecular replacement using *Trichodesmium erythraeum* FutA (TeFutA) (PDB ID: 6G7N) as the search model (Polyviou et al. 2018) and the refined apo McFbpA structure was used for building the holo McFbpA structure. Statistics for the data sets and the crystallographic models are presented in Tables S1 and S2, respectively.

**Fig. 4:**
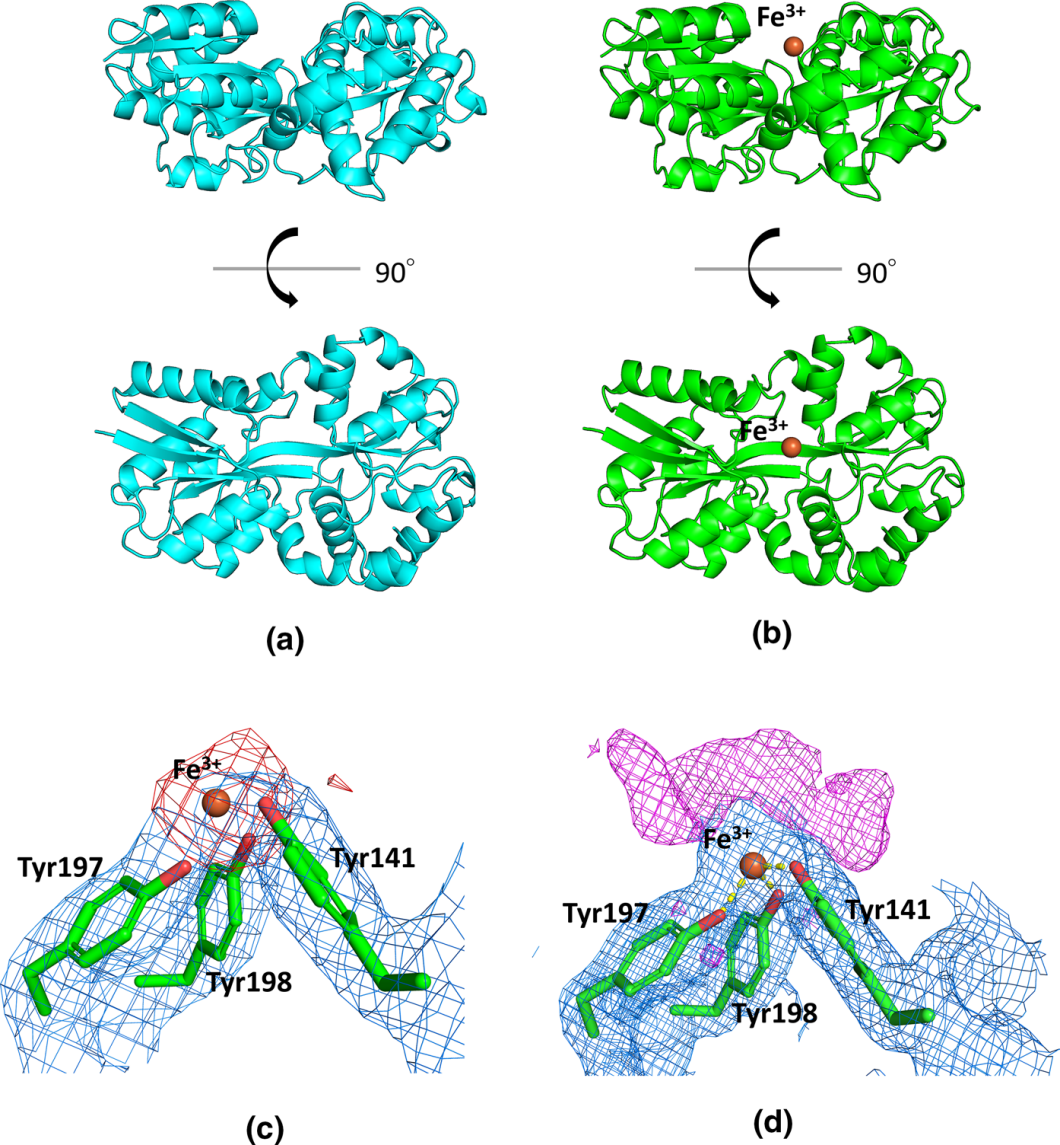
Crystal structures of McFbpA. Overall structures of **a** the iron-apo (cyan) and **b** the iron-holo (green) McFbpA. The ferric ion in the iron-holo McFbpA is shown as an orange sphere. **c** The anomalous difference Fourier map contoured at 4 σ is shown as red mesh in the iron-holo open McFbpA, with an orange sphere representing the ferric ion. The 2mFo-DFc electron density map is shown in blue mesh, contoured at 1 σ. **d** The ferric ion bound in the iron-holo open McFbpA and the surrounding unresolved density (mFo-DFc map contoured at 3 σ shown in magenta mesh). The 2mFo-DFc electron density map is shown in blue mesh, contoured at 1 σ. The ferric ion is displayed as the orange sphere. (Color figure online)

**Fig. 5:**
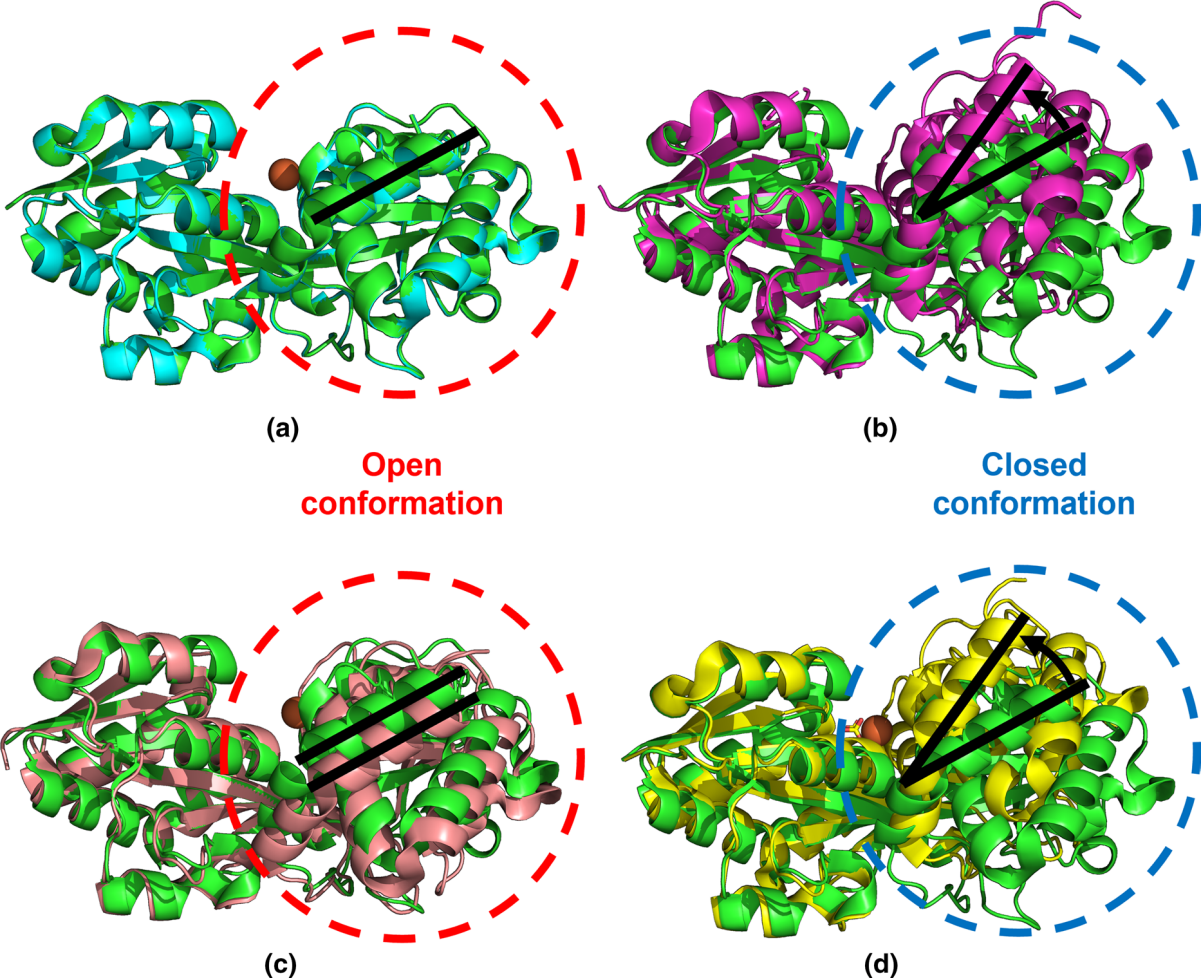
Structural alignment of the N-lobe alpha carbons from the *M. catarrhalis* apo FbpA (green) **a** with the N-lobe alpha carbons from *M. catarrhalis* holo FbpA (cyan) that is in the open conformation—top left, **b** with the *M. haemolytica* apo FbpA in a closed conformation (magenta, PDB ID 1Q35)—top right, **c** with the *M. haemolytica* holo FbpA in an open conformation (salmon, PDB ID 1SI1)—bottom left, and **d** with the *M. haemolytica* holo FbpA in a closed conformation (yellow, PDB ID 1SI0). The movement of the C-lobe in relation to the N-lobe is shown by the black lines and arrows. The red circle shows the comparisons with the open conformation and the blue circle shows the comparisons with the closed conformation. (Color figure online)

Similar to other FbpA structures, McFbpA has two globular α/β domains named the N terminal lobe (Asn1-Trp95 and Val235-Val273) and the C terminal lobe (Tyr107-Ala221 and Thr274-Lys312) (Fig. 4a, b). Each of the lobes consists of α helices surrounding a twisted mixed β sheet in the center. These two lobes are connected by a pair of antiparallel β strands (Phe96-Phe106 and Gly222-Val234), which acts as a hinge allowing for the adoption of either the open or closed conformation. The binding cleft lies between the globular domains and is lined with basic residues, which have synergistic anion binding function in other proteins of this class. Comparisons with other FbpAs indicate that both iron-holo and apo McFbpA in this study are in the open conformation (Fig. 5). The tyrosine residues (Tyr141, Tyr197 and Tyr198) are positioned in the same orientation as in other known structures, which classifies McFbpA as a class III iron-binding protein. The Fe–OH distances of Tyr141, Tyr197 and Tyr198 are 2.1 Å, 1.9 Å, and 2.1 Å, respectively. Analysis of electron density typical for metal ions, as illustrated in Fig. 4c, localizes the iron atom in the appropriate position. The electron density illustrated in Fig. 4d, likely represents components of the protein preparation or crystallization buffer that are interfering with FbpA achieving the closed conformation.

### Site-directed mutagenesis of iron binding tyrosine residues of *M. catarrhalis* FbpA

Based on identification of the iron-binding residues, Tyr141, Tyr197, and Tyr198, in the iron-holo McFbpA structure, we made site-directed mutations of these residues and reintroduced the *fbpA* gene with single residue mutations back into the Δ*fbpA* mutant to determine their impact on iron uptake from Tf and Lf. Site-directed mutations of FbpA were made through SOE-PCR, mutating each tyrosine residue into an alanine residue. The *fbpA* gene encoding the iron binding residue mutant and an aminoglycoside-3′-phosphotransferase gene were inserted between two ~ 600 bp regions that are upstream and downstream of the open reading frame via SOE-PCR, allowing for *fbpA* to be reintroduced into the bacterial genome via homologous recombination (Fig. 2).

Using the modified disk feeding assay, we determined that both the wildtype *M. catarrhalis* strain (N141) and the wildtype FbpA complemented strain (N509) were able to utilize human Tf and human Lf as iron sources. Porcine Tf was used as a negative control since the receptor proteins a highly host specific. All three iron binding residue mutants, Tyr141Ala (N515), Tyr197Ala (N519), and Tyr198Ala (N517), did not grow on either human Tf or human Lf (Table 2) indicating that they are essential for iron acquisition.

**Table 2:** Growth phenotypes of FbpA iron-binding residue mutants on BHI plus 50 μg/mL Desferal with hTf or hLf as sole iron source

	Wildtype (N141)	Δ*fbpA* (N404)	*Cis*-complemented strain (N509)	FbpA Tyr141Ala (N515)	FbpA Tyr197Ala (N519)	FbpA Tyr198Ala (N517)
Human Tf	+	−	+	−	−	−
Human Lf	+	−	+	−	−	−
Porcine Tf	−	−	−	−	−	−

5 μL of buffer alone or 3.1 nmol of hTf, hLf, or pTf, was spotted onto the plate

+ , Denotes growth; − , denotes no growth

### Homology modelling of *M. catarrhalis* AfeA

**Fig. 6:**
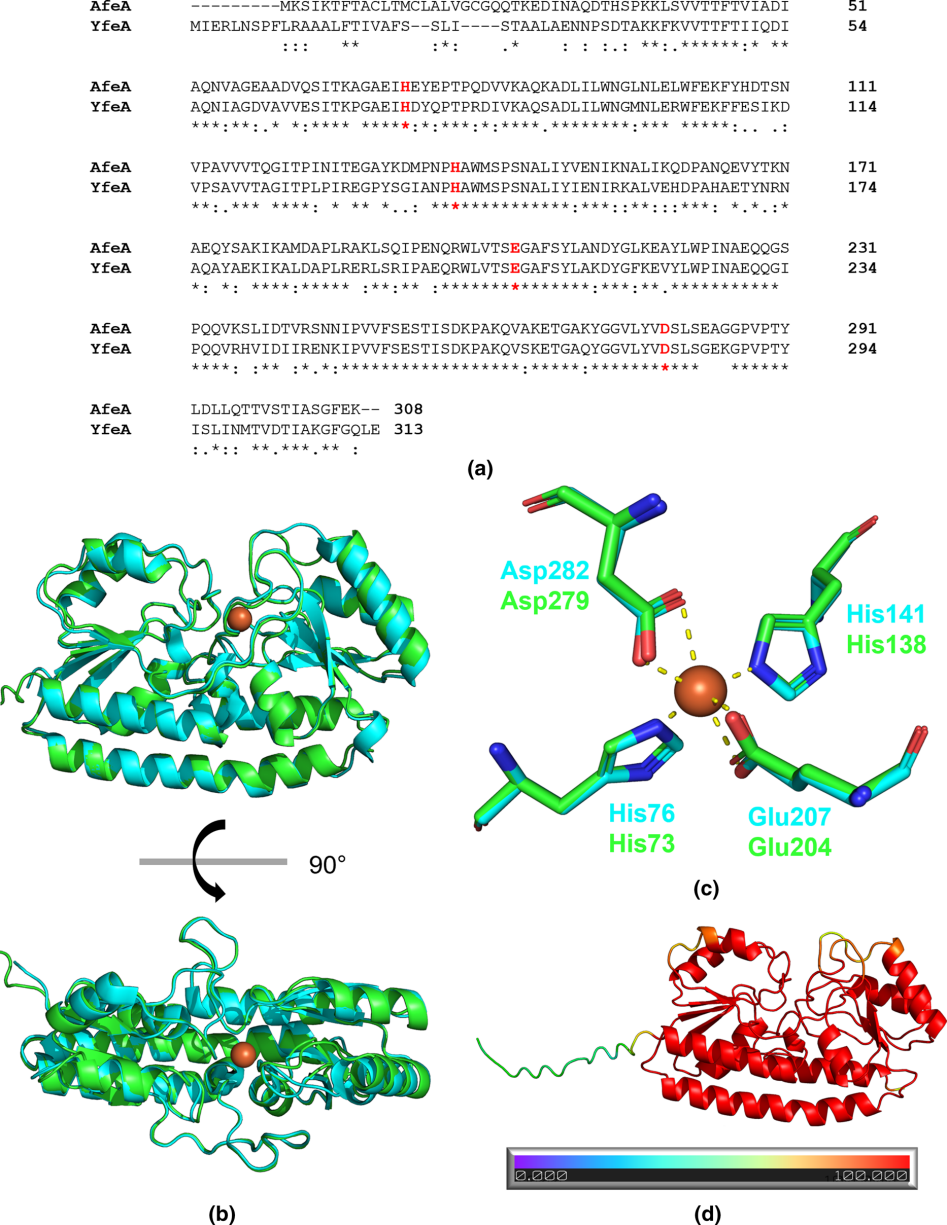
**a** Multiple sequence alignment of the *Y. pestis* YfeA and *M. catarrhalis* AfeA generated by Clustal Omega. Iron binding residues shared between YfeA and AfeA are shown in red letters. **b** Structural alignment between *Y. pestis* holo YfeA structure (cyan, PDB ID 5UY4) and homology model of *M. catarrhalis* AfeA (green) obtained from AlphaFold2. **c** Iron binding residues of YfeA that are shared with AfeA. Ferric ion is shown by an orange sphere and the yellow dashed lines show the interactions between the ferric ion and the binding residues. **d** Evaluation of model confidence of the AfeA homology model. Per-residue confidence score (pLDDT) were plotted on the homology model with a higher score meaning higher confidence. (Color figure online)

Sequence alignment of *M. catarrhalis* AfeA and *Y. pestis* YfeA revealed a 70% sequence identity and the conservation of metal binding residues that have been shown to bind to metal cations in YfeA (Fig. 6a). Using AlphaFold2, we were able to model *M. catarrhalis* AfeA. Structural alignment of the *M. catarrhalis* AfeA model to the *Y. pestis* holo YfeA crystal structure (PDB ID: 5UY4) shows a striking resemblance between the two. *Moraxella catarrhalis* AfeA adopts an overall fold of a C-clamp SBP with two domains. Each of the domains consists of a four stranded parallel beta sheet core surrounded by four alpha helices. The two domains of AfeA are connected through a rigid alpha helical backbone, which along with its metal-binding function classifies it as a cluster A-I SBP (Scheepers et al. 2016). A binding cleft is located between the two domains (Fig. 6b). The metal binding residues in the AfeA model (two histidines, one aspartate and one glutamate, Fig. 6c) are in identical positions in the binding cleft as the residues in the holo YfeA structure. Thermal shift assays (Murphy et al. 2017) demonstrate that the *M. catarrhalis* AfeA is polyspecific, able to bind ferric, ferrous, manganese, and zinc ions. This is analogous to what has been demonstrated for YfeA (Radka et al. 2017), indicating that AfeA and YfeA likely share the same mode of binding.

## Discussion

*Moraxella catarrhalis* is a Gram-negative diplococcus that colonizes the human nasopharynx, pharynx, conjunctiva, and genital tract. It is an opportunistic pathogen that causes bacterial otitis media (OM) in young children and exacerbates chronic obstructive pulmonary disease (COPD) in adults (de Vries et al. 2009; Murphy and Parameswaran 2009). It occupies a similar niche as most members of the *Neisseriaceae*, *Pasteurellaceae* and *Moraxellaceae* families and shares the ability to acquire iron directly from the host iron binding proteins (Tf and Lf) in humans and other mammals (Morgenthau et al. 2013). A detailed understanding of the structural and functional features of mechanisms involved in the acquisition of iron from Tf and Lf (Fig. 1) has been acquired from extensive studies in the pathogenic *Neisseria* species (Yadav et al. 2019; Noinaj et al. 2012b, 2013) and applies to other species that possess this uptake pathway. However, the known mechanisms cannot readily account for the observed reduction in growth dependent on exogenous Tf or Lf by loss of CopB in *M. catarrhalis* and IrpA of *M. bovis*.

In this study, we attempted to identify the periplasmic binding protein pathways that mediate transport of inorganic (non-siderophore bound) iron into the cell so that we would have a more complete understanding of the iron transport pathways in *M. catarrhalis.* Our approach using BLAST searches with known iron-binding periplasmic proteins identified only two periplasmic binding proteins that were homologues of the *M. haemolytica* FbpA (MhFbpA) (Shouldice et al. 2004, 2003b; Kirby et al. 1998) and the *Y. pestis* YfeA (Bearden et al. 1998). Using a combination of structural and functional studies we subsequently explored their potential role in iron acquisition from different iron sources.

**Fig. 7:**
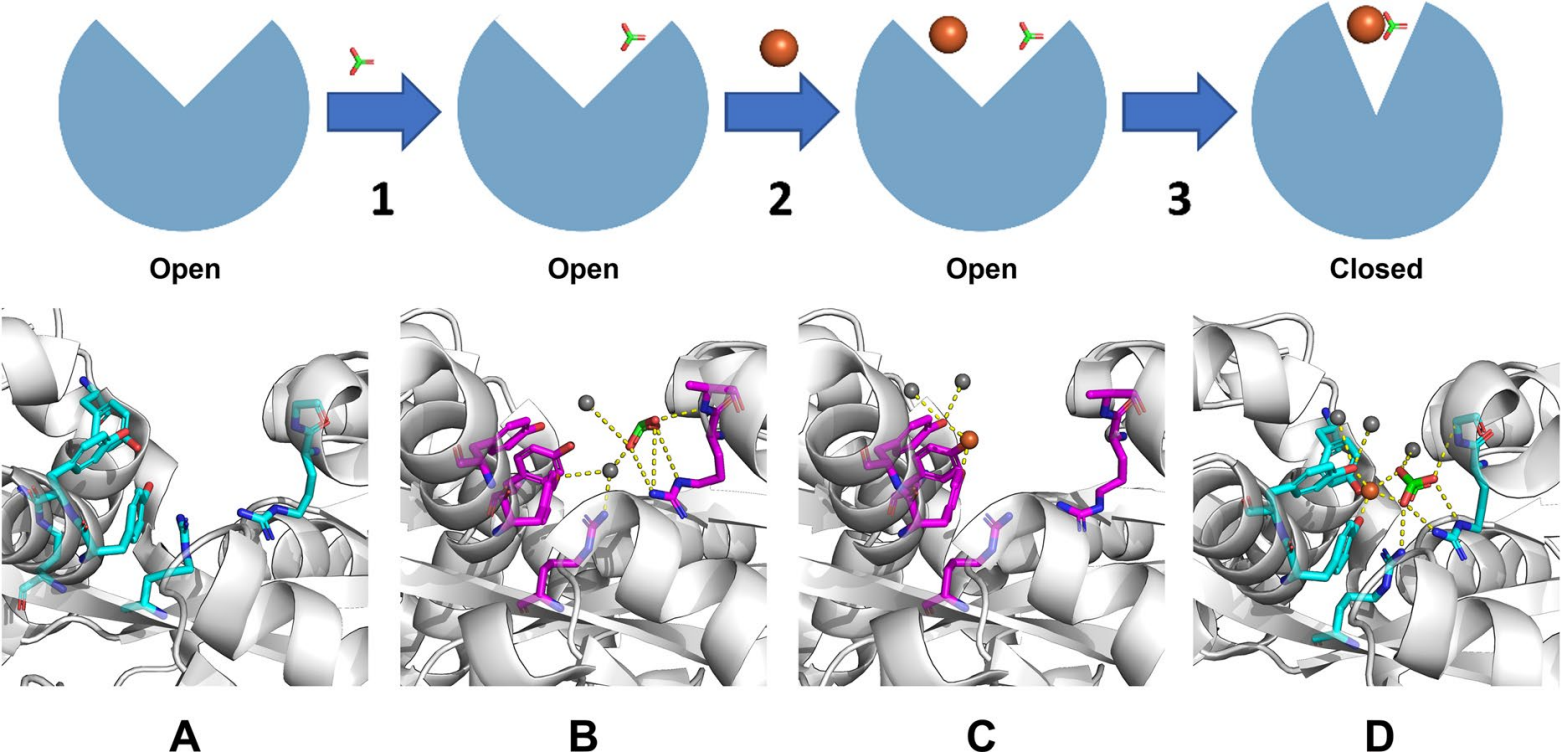
Proposed mechanism of FbpA ferric ion binding. (1) Carbonate binds to the iron-apo open FbpA. (2) Ferric ion binds to the three tyrosine residues, forming the iron-holo open FbpA. (3) FbpA adopts the closed conformation (Wang et al. 2011). Iron and carbonate binding residues from TtFbpA structures (Panels **A** and **D**) (PDB ID 4ELO and 4ELR) are shown as cyan sticks and from McFbpA structures (Panels **B** and **C**) (PDB ID 7LI0 and 7LI1) are shown as magenta sticks. Iron atoms are shown in orange spheres, water molecules are shown in grey spheres, and carbonate molecules are shown in green sticks. (Color figure online)

Our structural studies yielded the structure of *M. catarrhalis* FbpA in two different forms: an apo open form and an iron-holo open form. Like MhFbpA and *Thermus thermophilus* FbpA (TtFbpA), McFbpA adopts a typical fold of class III periplasmic iron binding protein with the two globular lobes. The studies with MhFbpA suggested that in contrast to the six liganding residues in Tf, Lf and other periplasmic iron binding proteins, there are only five coordinating ligands in a class III FbpA, the three tyrosine ligands and two contributed by the synergistic carbonate anion (Shouldice et al. 2004). The process of how iron is bound in a class III FbpA has been proposed in studies with *T. thermophilus* FbpA (TtFbpA) (Wang et al. 2011). The proposed mechanism of iron binding with insights from both the previous study and the current study is illustrated in Fig. 7. The synergistic anion (e.g., carbonate) binds to the open FbpA through the arginine residues in N terminal lobe (Fig. 7, Step 1). The ferric ion then binds to the three tyrosines in the C terminal lobe (Step 2) which results in the FbpA adopting a closed conformation through bidentate binding of iron by the carbonate anion (Step 3) (Wang et al. 2011).

There is some debate whether the carbonate mediates the closing of the protein. It was found in MhFbpA that the presence of a formate or carbonate in binding cleft favors the closed conformation, while the absence of carbonate favors the open conformation (Shouldice et al. 2004). The adoption of the closed conformation did not appear to be dependent on the presence of ferric ion. Based on this, it was concluded that the binding of a synergistic anion causes the adoption of the closed conformation (Shouldice et al. 2004). The authors suggested that the transfer of iron from TbpA to FbpA involved protein interactions that may be responsible for the iron transfer to FbpA and the subsequent demonstration of a TbpA-FbpA interaction with the *N. gonorrhoeae* proteins is consistent with this proposal (Siburt et al. 2009).

For TtFbpA, it was suggested that the bound synergistic anion promotes the binding of the ferric ion and that the clamping of the two lobes of the protein reinforces the binding between carbonate and the ferric ion (Wang et al. 2013). Clearly structural studies do not provide information on molecular dynamics and there likely are continual dynamic movements that are stabilized in particular conformations by the bound entities.

In the structure of iron-holo open McFbpA, we have determined that there are three tyrosine residues binding to the ferric ion and have confirmed that these residues are essential for the bacteria’s ability to utilize Tf and Lf as an iron source. There is additional electron density around the ferric ion in the structure, but it is indistinct for fitting waters and ligands. This is similar to what was observed in the crystal structure of the iron-holo open MhFbpA (Shouldice et al. 2004). We believe that it might be multiple components from the crystallization condition that are non-specifically binding to the ferric ion, resulting in the electron density that is indistinct for any one component. In one of the four FbpA molecules in the holo crystal structure, we determined that two additional ferric ions are occupying the density surrounding the ferric ion bound by the three tyrosines (Fig. S2). This might be caused by insufficient washing of the iron-bound protein, allowing for a slight excess amount of unbound ferric ion to remain with the protein. This resembles the ferric hydroxide clusters that were found in the *H. influenzae* FbpA mutant proteins where site-directed mutations on different residues were made (Shouldice et al. 2003c).

Using AlphaFold2, we were able to produce a high-quality model of AfeA, which closely resembles *Y. pestis* YfeA (Fig. 6b). In the binding cleft, the two histidines, the aspartate, and the glutamate are positioned in a manner that allows for optimal ferric ion binding (Fig. 6c), which suggests that AfeA is similar to YfeA in its ability to bind metal cations, like iron. AfeA was shown to bind ferric, ferrous, manganese, and zinc ions with a thermal shift assay in a previous study (Murphy et al. 2017) thus it might play a role in transporting any or all of these metal cations.

The *M. catarrhalis* Fbp and Afe iron transport systems are reminiscent of the periplasmic molybdate transport in *H. influenzae* where there is a high affinity transport system, ModABC, and a low affinity transport system, MolABC. The structure of ModA is more similar to FbpA and both are known to bind to only one substrate. MolA, on the other hand, is a C-clamp protein like AfeA and both of these proteins are known to be polyspecific, binding more than one substrate (Murphy et al. 2017; Tirado-Lee et al. 2011). ModA has a significantly higher affinity to molybdate in the nM to low μM scale while MolA has a lower affinity in the 100 μM scale (Tirado-Lee et al. 2011). The ModABC system functions primarily when there are low concentrations of molybdate while the MolABC system is more efficient when there are high molybdate concentrations (Rice et al. 2014). This might explain the differences in growth phenotypes between the Δ*fbpA* mutant and the Δ*afeA* mutant. FbpABC is essential when using human Tf and Lf as iron sources, due to the low amount of iron, ~ 3.1 nM, that these iron sources provide, which requires a high affinity transport system. In addition, as described above, the transfer of iron from TbpA to FbpA may involve protein–protein interactions.

Being a low affinity transport system, AfeABC cannot transport the small amount of iron from these iron sources. The high affinity FbpABC transport system also appears to be essential when transporting lower concentrations of ferric citrate, ~ 25 nmol. The difference in the amount of iron required for growth between human Tf/Lf and ferric citrate might be due to the greater difficulty of Desferal sequestering iron from iron binding proteins as compared to ferric citrate. The AfeABC system does not seem to be important for transport of ferric citrate under the experimental conditions of this study. It is possible that we cannot discern the minute differences in growth on ferric citrate between the wildtype and the Δ*afeA* mutant using the feeding assay, as the presence of a high affinity transport system would overshadow these small differences. In the context of the human host, we believe FbpA would have a greater role in iron uptake than AfeA, as the concentration of ferric ion in the host environment is low and human Tf/Lf are the iron sources that are accessible for the bacteria.

Based on the protein sequence, AfeA was previously predicted to be a lipoprotein and evidence for surface exposure was provided by whole-cell ELISA and flow cytometry (Murphy et al. 2017). This raises questions regarding the role of AfeA in mediating metal ion transport. Is it involved in initial capture of extracellular metals ions or does it function in mediating the transport of metal ions across the periplasmic space? If AfeA is indeed transported to the surface of the outer membrane, an important question is how it is able to get there. The only known export system for SLPs in the bacterial species being considered is the SLAM system (Hooda et al. 2016, 2017). As AfeA shares no sequence similarity to other SLPs, it is unlikely to be a substrate for SLAM-mediated export. It is also salient to note that there were several published studies reporting that *Neisseria* FbpA is surface exposed (Gómez et al. 1996, 1998), a conclusion not shared by many in the field. Notably *Neisseria* FbpA was initially proposed to be an outer membrane protein (Mietzner et al. 1984), but subsequently shown to function in periplasm to cytoplasm transport of iron (Chen et al. 1993) prior to the studies on surface exposure. Since the periplasmic role and location of FbpA has been widely accepted, and there is no evidence for transport of FbpA to the surface, it raised the question of how to explain the experimental results. One possible explanation is that FbpA may be released by cell lysis or leakage from the periplasmic space and is subsequently associated with the cell surface, and an important question is whether this also occurs under in vivo conditions.

**Fig. 8:**
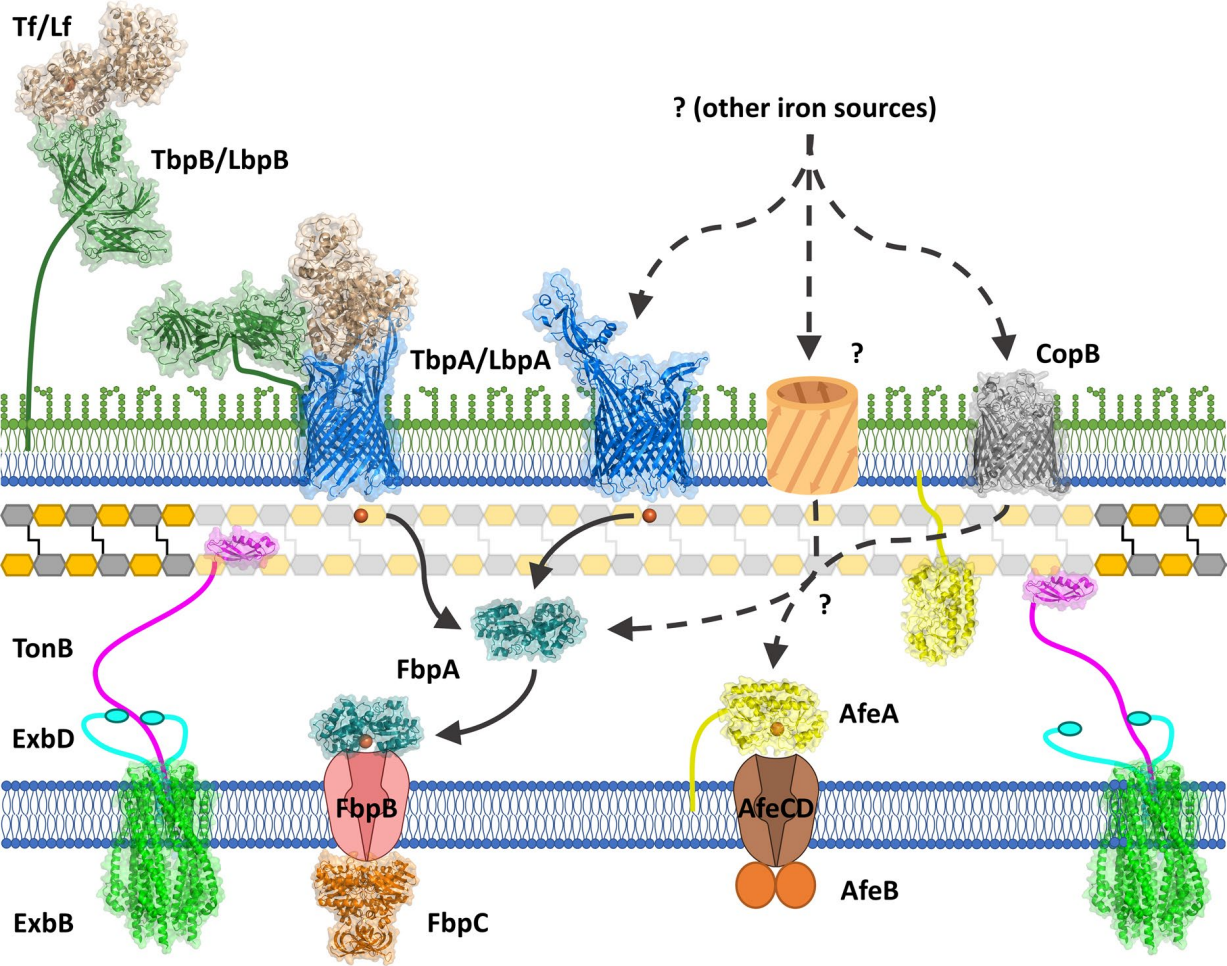
Transport of iron from various iron sources through the bacterial cell envelope in *M. catarrhalis*. Iron from transferrin and lactoferrin is extracted by the TbpB-TbpA or LbpB-LbpA complex and transported across the membrane with energy provided by TonB. The iron is then transported by the FbpABC periplasm to cytoplasm pathway into the cell. The FbpABC pathway is also responsible for transporting iron from other sources present in BHI media but which outer membrane system(s) are involved has not been determined. Similarly, the AfeABCD pathway is required for transport of other iron sources that are present in more complex media but how they are transported across the outer membrane is uncertain

The presence of a ‘lipobox’ in AfeA distinguishes it from YfeA, but its presence in a typical periplasm to cytoplasm transport operon strongly argues for its role in delivering metal ion to the inner membrane transport proteins (Fig. 8). It has not been demonstrated whether AfeA remains associated with the inner membrane or is transported to the inner leaflet of the outer membrane by the Lol system (Yokota et al. 1999) but since it has a reasonably long anchoring peptide (20 amino acids) it could function in the periplasmic metal ion transport tethered to either membrane. The key question is which TBDT or transporters it serves for metal ion transport across the periplasmic space (Fig. 8) or whether it is part of a novel system for metal ion transport that is independent of TBDTs.

The structures and metal binding properties of various classes of metal ion binding proteins have been well characterized. However, a role in transporting iron derived from host Tf and/or Lf has only been established with FbpAs from *N. meningitidis* and *H. influenzae*, which all belong to the class I iron-binding protein family (Khun et al. 1998; Kirby et al. 1997). In this study we wanted to determine whether McFbpA, a class III iron-binding protein, or AfeA, a cluster A-I SBP metal-binding protein, were involved in the transport of iron obtained from Tf and Lf. We attempted to prepare deletion mutants of these two proteins and were able to isolate a Δ*afeA* mutant on BHI medium but were only successful in obtaining a Δ*fbpA* mutant by selection on chocolate plates. The results indicate that without McFbpA the iron sources in BHI were insufficient to support the growth required to form colonies, clearly demonstrating that McFbpA is involved in acquisition of iron from sources other than Tf and Lf (Fig. 8). We were unable to obtain Δ*fbpA*Δ*afeA* mutant on chocolate plates, suggesting that the two metal binding proteins acquire metal ions from different sources (Fig. 8) and collectively are a lethal mutation even when provided with a very rich source of metal ions. Notably, we were able to introduce non-functional iron-binding FbpA mutants into the Δ*fbpA* mutant, showing that the ability of the Δ*fbpA* mutant to take up exogenous DNA and incorporate it into the genome is unaltered and that it is the lethality of knocking out both *fbpA* and *afeA* that prevented us from obtaining Δ*fbpA*Δ*afeA* mutant colonies.

The results from the modified disk feeding assay demonstrate that the Δ*fbpA* mutant is unable to grow on human Tf and Lf whereas the Δ*afeA* mutant is unaffected (Fig. 3). This indicates that the iron released by TbpA and LbpA in the periplasm must be bound by FbpA for transport across the periplasm to the cytoplasmic membrane where the FbpBC complex mediates its transport across the inner membrane into the cytoplasm (Fig. 8). Hydrogen/deuterium exchange studies have indicated that there is close association between FbpA and TbpA during the transfer of iron in *N. gonorrhoeae* (Siburt et al. 2009) which could be essential for efficient transfer. To what extent this observation applies to other TBDTs to achieve efficient coupling of the transport across the outer membrane and the subsequent transport across the periplasm and into the cell is currently unknown. Although this type of interaction may not be necessary for capture of soluble substrates such as iron–siderophore complexes or iron complexed to organic acids, the release of bound ferric ion by TbpA, LbpA or other TBDTs to the periplasmic binding proteins may be essential.

The inability of the Δ*fbpA*Δ*afeA* strains to grow on chocolate plates while the individual deletion mutant strains were able, suggests that the two periplasmic metal binding proteins service uptake pathways for iron. Notably, in the modified disk feeding assay (Fig. 3) the excess ferric citrate applied to the wild type cells plated on media containing the ferric ion chelator, Desferal, is not completely complexed which results in growth dependent upon the concentration of ferric citrate. There is no detectable impact on growth by the Δ*afeA* strain but a detectable impact on growth by the Δ*fbpA* strain, suggesting that McFbpA has a more significant role to play in transport of iron from ferric citrate. Previous studies with a Δ*afeA* strain revealed a reduced growth rate on chemically defined medium (Murphy et al. 2016; Juni et al. 1986) that was interpreted as a deficiency in transport of ferric ion. Taken together with the results in this study it suggests that the nature of the ferric ion complexes will impact which uptake pathway will be involved but the degree to which this depends on the TBDT, the periplasm-to-cytoplasm pathway or the interaction between the periplasmic binding protein and TBDT is uncertain (Fig. 8).

In summary, this study has characterized the *M. catarrhalis* periplasmic iron transport structurally and functionally. Two high resolution crystal structures of the *M. catarrhalis* FbpA were solved in the iron-holo open and apo open conformations, revealing more details about the mechanism behind FbpA-mediated iron transport. Isogenic mutants of FbpA and AfeA were constructed, demonstrating that FbpA is essential for the utilization of human Tf and Lf as iron sources and that AfeA does not take up iron from these iron sources (Fig. 3). The inability to prepare a strain lacking both of these systems on such an iron rich source such as chocolate plates, suggests that there is no other periplasm to cytoplasm iron acquisition pathway in the strain of *M. catarrhalis* used in this study but that additional studies will be needed to try and determine the pathways for other iron sources across the outer membrane.

## Materials and methods

### Bioinformatics analysis

To compare the repertoire of iron acquisition proteins in *Moraxella* and *Neisseria* species, we performed BLASTP searches with the organism limited to *M. catarrhalis*, *M. bovis*, *N. meningitidis*, or *N. gonorrhoeae*. *Moraxella catarrhalis* CopB, FbpA, AfeA, and *N. gonorrhoeae* FetB were used as search queries (Altschul et al. 1997). All other parameters were left as default. The top hits with the lowest BLAST *E-*value were used for comparison in Table 1.

To identify putative periplasm-to-cytoplasm transport pathways for ferric ion in *M. catarrhalis*, PSI-BLAST searches were performed with the organism limited to *M. catarrhalis* using the periplasmic iron-binding proteins from *H. influenzae* (FbpA: PDB ID 3OD7), *Y. pestis* (YfeA: PDB ID 5UY4), *Serratia marcescens* (SfuA: PDB ID 1XVY) and *M. haemolytica* (MhFbpA: PDB ID 1SI0) as search queries (Altschul et al. 1997). Default values were used for all other parameters. Hits with the lowest BLAST *E*-value were selected as putative homologs.

### Protein production and purification

*Moraxella catarrhalis* strain Q8 *fbpA* was cloned into a custom pT7-7 vector and the gene was expressed in *Escherichia coli* strain ER2566. The transformed colonies were first cultured overnight in LB broth with added 100 μg/mL of ampicillin. The starter culture was then diluted 1:1000 in 6 L of autoinduction media with added 100 μg/mL of ampicillin. The culture was grown at 37 °C for 24 h. The desired protein was purified from the periplasm using a modified osmotic shock method. The culture was centrifuged at 5000 × g for 30 min at 10 °C. The supernatant was decanted and the cell pellet was resuspended in 300 mL of osmotic shock buffer (30 mm Tris/HCl buffer pH 8.0, 20% sucrose, 1 mM EDTA). The cell mixture was then shaken for 10 min at room temperature and centrifuged at 3220 × g for 30 min. The supernatant was decanted and the cells were resuspended in 60 mL of ice cold 5 mM MgSO_4_. The resuspended cells were incubated on ice for 10 min and then were centrifuged at 3220 × g for 30 min. The osmotic shock fluid was carefully removed from the cell pellet and was dialyzed against the anion exchange equilibration buffer (50 mM Tris pH 8.0, 10 mM NaCl). The protein was then concentrated using a Vivaspin 20 with a 10 kDa cut-off (GE Healthcare) and injected into a 5 mL HiTrap Q HP column using an ÄKTA fast protein liquid chromatography system (GE Healthcare). The protein from the flow through was collected and was concentrated. The protein was washed with a citrate/bicarbonate buffer (100 mM sodium citrate pH 7.4, 100 mM sodium bicarbonate) and 5 M excess of ferric citrate was added to the protein. The mixture was incubated on ice for 30 min and was thoroughly washed with 10 mM Tris buffer pH 8.0. The protein was then concentrated to 50 mg/mL.

### Protein crystallization

Sitting drop vapour diffusion was used for all crystallization in this study. Initial crystallization screens were done using Hampton Index HT crystallization screen and setup using the NT8® Drop Setter. The crystals were grown at 298 K and appeared within a day of crystallization setup. A red–orange colour was observed with the holo FbpA crystals while apo FbpA appeared colourless. The apo FbpA crystals were grown in a 4 μL drop with 1:1 protein to reservoir ratio in a condition containing 0.1 M ammonium citrate tribasic pH 6.5, 30% v/v polyethylene glycol 3,350. The drop was then equilibrated against a 500 μL reservoir and the crystals were grown at 298 K for 7 days. The iron-holo FbpA crystals were grown in a 4 μL drop with 1:1 protein to reservoir ratio in a condition containing 0.2 M lithium sulfate monohydrate, 0.1 M HEPES pH 7.5, 30% v/v polyethylene glycol 3350. The drop was then equilibrated against a 500 μL reservoir and the crystals were grown at 298 K for 7 days.

### Crystal harvesting, data collection, structure solution and refinement

The crystals were harvested in a nylon loop and were washed in a cryoprotectant solution for ~ 15 s. The cryoprotectant for the apo FbpA crystals contained 0.1 M ammonium citrate tribasic pH 6.5, 30% v/v polyethylene glycol 3350, 30% v/v glycerol and the cryoprotectant for the holo FbpA crystals contained 0.2 M lithium sulfate monohydrate, 0.1 M HEPES pH 7.5, 35% v/v polyethylene glycol 3350. The crystals were then vitrified in liquid nitrogen and were maintained in a nitrogen stream at 100 K during data collection. The diffraction data for the apo FbpA crystals was collected at the National Synchrotron Light Source II (NSLS-II) 17-ID-1 beamline using an EIGER 9M detector at the wavelength of 0.92009 Å. The diffraction data for the holo FbpA crystals was collected at the Canadian Light Source (CLS) 08B1-1 beamline using a Rayonix MX300HE CCD X-ray detector at a wavelength of 1.0332 Å. An iron-single-wavelength anomalous dispersion (SAD) dataset was also collected at the same beamline at a wavelength of 1.7384 Å to empirically determine the presence of iron and its atomic coordinates (Table S1). Data reduction and scaling were done using iMOSFLM and AIMLESS for the apo FbpA dataset (Battye et al. 2011; Evans and Murshudov 2013), and XDS and XSCALE for the holo FbpA dataset (Kabsch 2010). The space group assignments of both structures were verified using the program Zanuda (Lebedev and Isupov 2014). The structures were solved by molecular replacement using Phaser from the PHENIX suite (Table S2). Model building was done using Autobuild from the PHENIX suite. Multiple iterations of refinement were done using phenix.refine from the PHENIX suite (Liebschner et al. 2019) and manual manipulations using COOT (Emsley and Cowtan 2004).

### Homology modelling

AlphaFold2 (Jumper et al. 2021) hosted on ColabFold’s online notebook (Mirdita et al. 2022) was used to generate homology models. The model quality was assessed using per-residue confidence score (pLDDT) (Mariani et al. 2013).

### Construction of the isogenic FbpA and AfeA knockouts

To delete the *fbpA* or *afeA* gene from the *M. catarrhalis* genome, a knockout construct that included the chloramphenicol acetyltransferase gene or the aminoglycoside-3′-phosphotransferase gene inserted between two ~ 600 bp upstream/downstream region was made through splicing by overlap extension PCR (SOE-PCR). A liquid culture of *M. catarrhalis* strain No. 17 (N141) was grown to an optical density of 0.4. 1 mL of the culture was harvested and centrifuged at 5939 × g for 1 min at 4 °C. The supernatant was decanted and the cell pellet was resuspended in 50 μL of the PCR reaction mixture. The resuspended cells were then spotted onto a chocolate agar plate that was incubated overnight at 37 °C supplemented with 5% CO_2_. The bacteria were resuspended in BHI and then plated at various dilutions on chocolate agar for the Δ*fbpA* mutant and BHI agar for the Δ*afeA* mutant, containing 1 μg/mL of chloramphenicol or 20 μg/mL of kanamycin respectively. The plates were incubated overnight at 37 °C supplemented with 5% CO_2_. Colonies containing the gene deletions were screened for and verified by PCR.

### Construction of FbpA iron binding residue mutants

To create *M. catarrhalis* strains expressing FbpA protein with single iron binding residue mutations, a knock-in construct was created for each iron binding residue mutation. The desired *fbpA* mutations were first made through SOE-PCR. Then, the knock-in construct containing the *fbpA* gene and the aminoglycoside-3′-phosphotransferase gene flanked by two ~ 600 bp upstream/downstream region was created through SOE-PCR. Natural transformation of the knock-in constructs into *M. catarrhalis* and the selection of desired colonies were performed as mentioned above. Colonies containing the mutant *fbpA* genes were screened for and verified by PCR and Sanger sequencing.

### Feeding assay

Bacterial strains were grown in BHI broth overnight at 37 °C in a shaking incubator. The liquid culture was then diluted with BHI to an OD of 0.05 and 100 μL of the diluted culture was used to inoculate BHI plates containing 50 μg/mL of desferrioxamine mesylate or Desferal (Sigma). Tf and Lf were prepared by dissolving holo human Tf (Sigma), holo human Lf (Sigma), or holo porcine Tf (Gibco) in 50 mM HEPES pH 8.0, 50 mM NaCl. Haemin was prepared by incubating 10 mg in 10 mL of 4% v/v triethanolamine. 5 μL of each of the iron sources was spotted onto the desferrated BHI agar plate that was incubated at 37 °C with 5% CO_2_.

## Supplementary Information

Below is the link to the electronic supplementary material.

Supplementary file1 (DOCX 2381 kb)

## Data Availability

The raw data supporting the conclusions of this article will be made available by the authors, without undue reservation.
