# Supplementary material for: Structural and functional insights into iron acquisition from lactoferrin and transferrin in Gram-negative bacterial pathogens
Source: Biometals. 2022 Nov 23;36(3):683–702. doi: 10.1007/s10534-022-00466-6 (PMC10182148; doi:10.1007/s10534-022-00466-6)
Supplement: Supplementary file 1 — Supplementary file1 (DOCX 2381 kb) [file 10534_2022_466_MOESM1_ESM.docx]

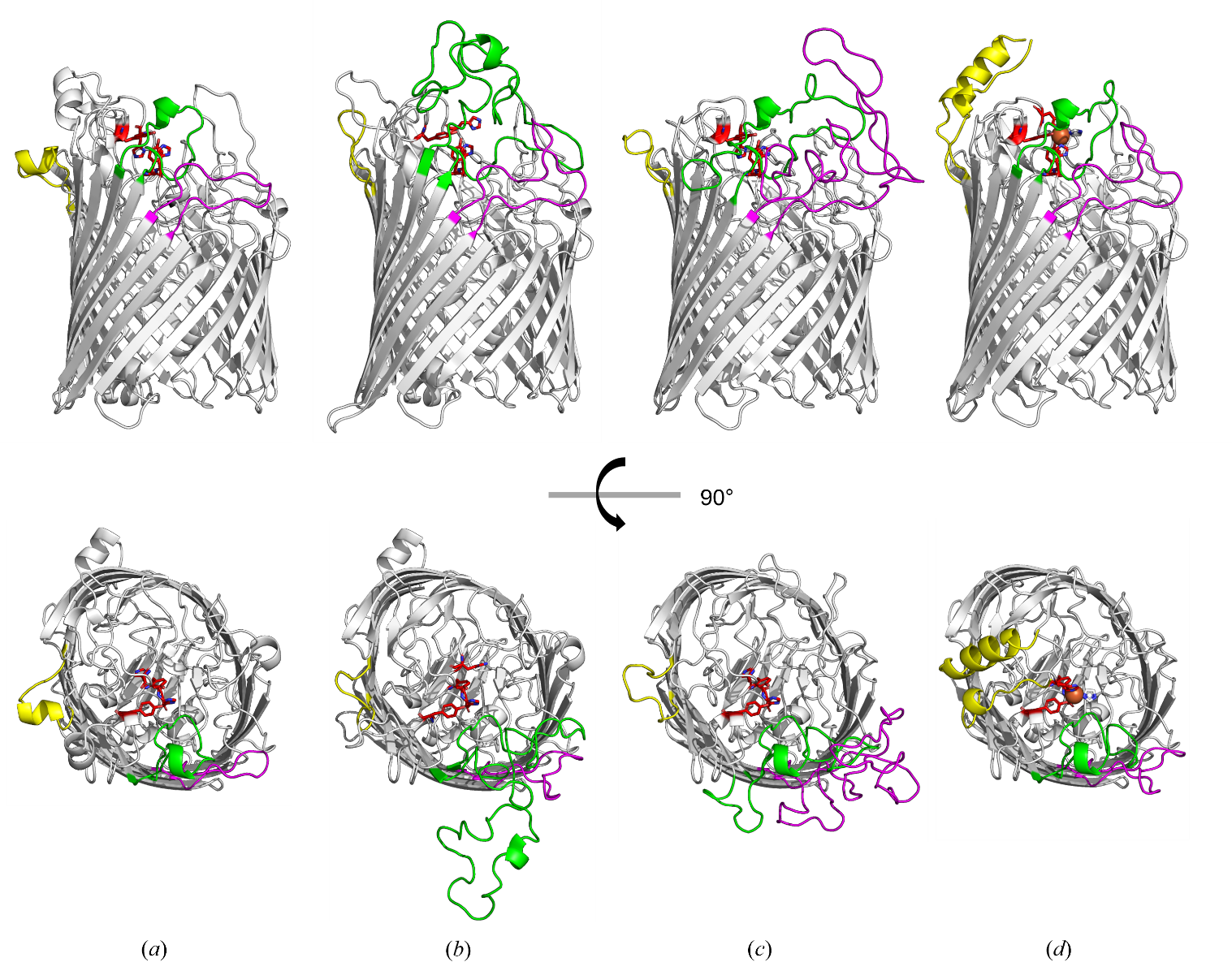


**Figure S1.** Homology models of *M. catarrhalis* CopB from strains 417:082 (*a*) and O35E (*b*), and *M. bovis* IrpA (*c*). Crystal structure of iron-holo *N. meningitidis* FetA (PDB: 4AIQ) (*d*). Loop 2 is coloured in magenta, loop 3 is coloured in green, and loop 5 is coloured in yellow. Conserved iron binding residues are shown as red sticks. Iron atom is shown as orange sphere.


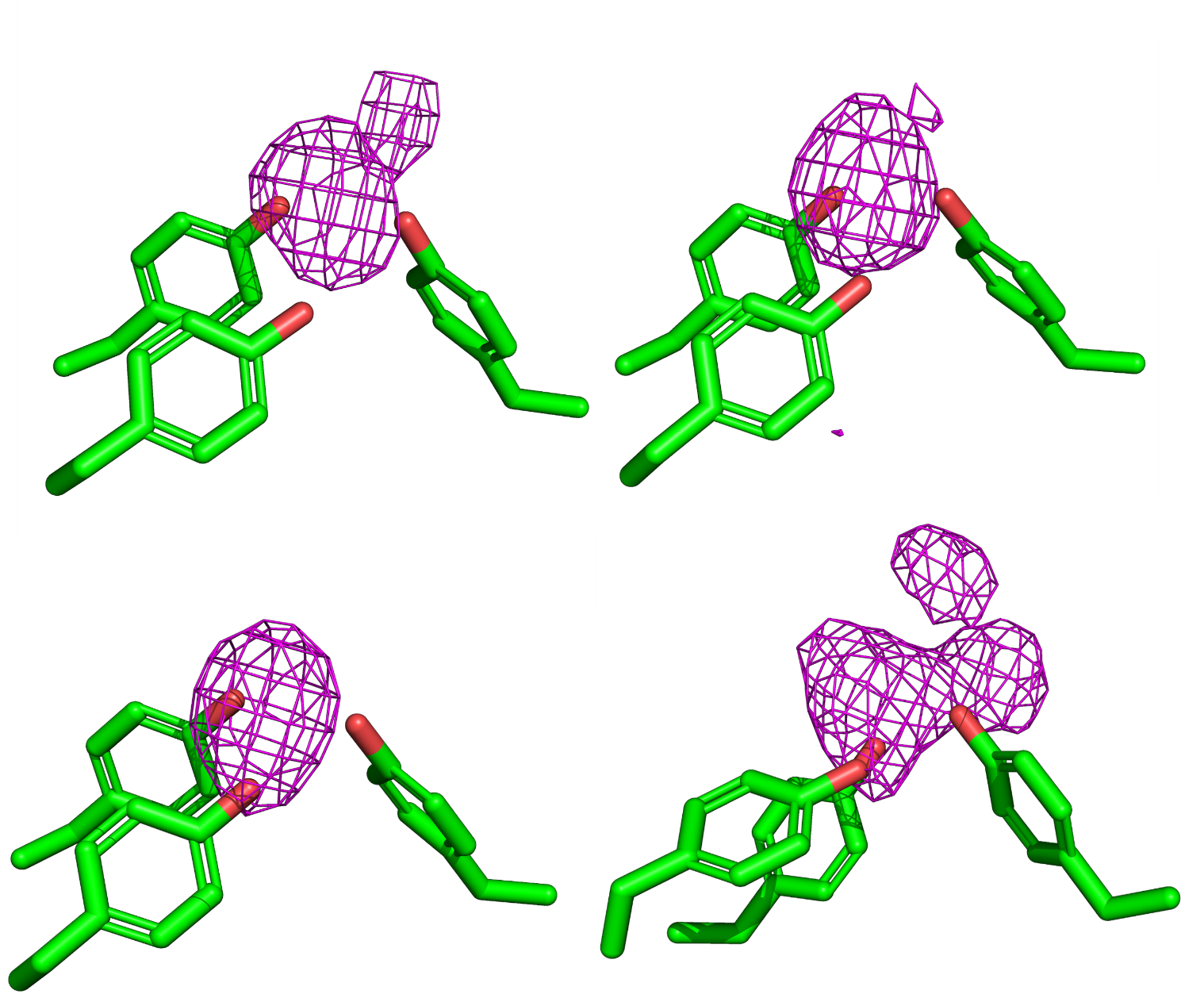


**Figure S2.** Anomalous difference Fourier maps of the four iron binding sites of iron-holo open McFbpA (green) contoured at 4 σ (magenta mesh).

**Table S1.** Data collection and processing.

Values for the outer shell are given in parentheses.

|  | Apo McFbpA | Holo McFbpA | |
| --- | --- | --- | --- |
| PDB Identifier | 7LI0 | 7LI1 |  |
| Diffraction source | NSLS-II 17-ID-1 | CLS 08B1-1 | CLS 08B1-1 |
| Wavelength (Å) | 0.92009 | 1.03321 | 1.7384 |
| Temperature (K) | 100 | 100 | 100 |
| Detector | EIGER 9M | Rayonix MX300HE | Rayonix MX300HE |
| Crystal-detector distance (mm) | 220 | 200 | 119.20 |
| Rotation range per image (°) | 0.20 | 0.50 | 0.25 |
| Total rotation range (°) | 180 | 180 | 360 |
| Exposure time per image (s) | 0.05 | 0.5 | 1.0 |
| Space group | *P*1 | *P*1 | *P*1 |
| *a*, *b, c* (Å) | 62.06, 67.83, 73.53 | 62.13, 67.56, 73.77 | 62.06, 67.54, 73.74 |
| *α*, *β*, *γ* (°) | 91.28, 91.59, 105.82 | 91.02, 92.26, 105.02 | 91.00, 92.24, 105.04 |
| Mosaicity (°) | 0.26 | 0.26 | 0.35 |
| Resolution range (Å) | 51.61-1.84 (1.87-1.84) | 29.71-1.75 (1.81-1.75) | 30.14-2.35 (2.43-2.35) |
| Total no. of reflections | 172012 (7840) | 219684 (21800) | 173371 (16778) |
| No. of unique reflections | 96783 (4682) | 110222 (10935) | 45904 (4406) |
| Completeness (%) | 96.8 (93.8) | 94.25 (93.44) | 95.21 (92.24) |
| Multiplicity | 1.8 (1.7) | 2.0 (2.0) | 3.8 (3.8) |
| 〈*I*/*σ*(*I*)〉^#^ | 6.6 (1.7)  1.88 Å | 16.74 (0.83)  1.92 Å | 16.51 (1.25)  2.43 Å |
| *R*_merge._ | 0.058 (0.416) | 0.023 (1.01) | 0.048 (1.494) |
| CC_1/2_ | 0.992 (0.795) | 1.00 (0.424) | 0.999 (0.761) |
| Overall *B* factor from Wilson plot (Å^2^) | 24.56 | 33.76 | 53.47 |
| * The resolution limit was determined using CC_1/2_. The resolutions at which 〈*I*/*σ*(*I*)〉 falls below 2.0 are listed. | | | |

**Table S2**. Structure solution and refinement.

Values for the outer shell are given in parentheses.

|  | Apo McFbpA | Holo McFbpA |
| --- | --- | --- |
| PDB Identifier | 7LI0 | 7LI1 |
| FbpA in ASU | Four | Four |
| Ligand(s) | CIT, PEG, GOL, NA, CO3 | FE, CO3 |
| Resolution range (Å) | 51.61-1.85 (1.92-1.85) | 29.71-1.75 (1.81-1.75) |
| Completeness (%) | 96.67 (94.21) | 94.25 (93.40) |
| No. of reflections, working set | 95165 (9277) | 110042 (10906) |
| No. of reflections, test set | 1725 (159) | 2000 (205) |
| Final *R*_cryst_ | 0.1932 (0.2890) | 0.2311 (0.4171) |
| Final *R*_free_ | 0.2229 (0.3071) | 0.2650 (0.4302) |
| Number of non-H atoms | | |
| Protein | 9399 | 9329 |
| Ligand | 48 | 38 |
| Water | 735 | 373 |
| Total | 10182 | 9740 |
| Rms deviations from ideal values | | |
| Bonds (Å) | 0.004 | 0.006 |
| Angles (°) | 0.65 | 0.74 |
| Average temperature factors (Å^2^) | | |
| Protein | 34.25 | 39.88 |
| Ligand | 58.40 | 48.93 |
| Water | 38.52 | 38.80 |
| Ramachandran plot (%) | | |
| Favoured regions | 98.87 | 99.03 |
| Allowed | 1.05 | 0.97 |
| Outliers | 0.08 | 0.00 |
